# The Anti‐Leukemic Potential of Curcumin in Chronic Myeloid Leukemia: A Systematic Review of In Vitro Studies

**DOI:** 10.1002/fsn3.70852

**Published:** 2025-09-07

**Authors:** Hossein Bahari, Zeinab Salim, Leila Kardanpour, Hosein Kouchaki, Fatemeh Shoja, Iman Rahnama, Arezoo Faridzadeh, Maryam Ranjbar Zahedani

**Affiliations:** ^1^ Department of Nutrition, Faculty of Medicine Mashhad University of Medical Sciences Mashhad Iran; ^2^ Department of Nutrition Sciences, School of Health Larestan University of Medical Sciences Iran; ^3^ Shiraz Institute for Cancer Research, School of Medicine Shiraz University of Medical Sciences Shiraz Iran; ^4^ Student Research Committee Mashhad University of Medical Sciences Mashhad Iran; ^5^ Immunology Research Center Mashhad University of Medical Sciences Mashhad Iran

**Keywords:** apoptosis, cell proliferation, chronic myeloid leukemia, curcumin, drug resistance

## Abstract

Chronic myeloid leukemia (CML), a myeloproliferative neoplasm, is characterized by the *BCR‐ABL1* fusion gene, which results in constitutive tyrosine kinase activity. While tyrosine kinase inhibitors (TKIs) have significantly improved CML outcomes, resistance and the persistence of leukemic stem cells remain major clinical challenges. Curcumin, a natural polyphenol derived from *Curcuma longa* , has demonstrated potential anticancer properties. This review explores curcumin's effects on CML cell lines, focusing on its mechanisms of action and therapeutic potential. A systematic literature search was conducted in December 2024 across PubMed, Scopus, and Web of Science databases, following Preferred Reporting Items for Systematic Reviews and Meta‐Analyses (PRISMA) guidelines. The review included original in vitro studies examining curcumin's anti‐leukemic effects on human CML cell lines. Data was extracted and synthesized narratively due to methodological variability. Of 869 screened articles, 21 in vitro studies met the inclusion criteria. Curcumin inhibited proliferation and induced apoptosis in CML cell lines, particularly K562. Key mechanisms included inhibition of protein kinase C alpha (PKCα), Wilms' tumor 1 (WT1), *BCR‐ABL1* signaling, and histone deacetylase 8 (HDAC8) expression, as well as modulation of microRNA‐21 (miR‐21), phosphatase and tensin homolog (PTEN), and suppressor of cytokine signaling 1 and 3 (SOCS1/3) pathways. Curcumin also triggered cell cycle arrest at the G2/M phase and promoted autophagy and mitochondrial dysfunction. Notably, curcumin derivatives such as pentagamavunon‐1 (PGV‐1) and compound 1206 (C1206) displayed enhanced potency and overcame resistance in imatinib‐resistant CML cells. Curcumin demonstrates multi‐targeted anti‐leukemic activity in vitro, disrupting oncogenic signaling, epigenetic regulation, and redox balance in CML cells. Its ability to sensitize resistant cells and enhance apoptotic pathways positions it as a promising adjunct to current CML therapies. However, clinical translation requires further investigation to overcome pharmacokinetic limitations and validate efficacy in vivo.

## Graphical abstract

Curcumin suppresses chronic myeloid leukemia progression through multiple molecular pathways. It inhibits BCR‐ABL1, modulates miR‐21, PKCα, and PTEN, activates DR4/DR5, blocks G2/M cell cycle progression, and inhibits HDAC. These actions collectively promote apoptosis, enhance tumor suppressor expression, and reduce leukemic cell proliferation.

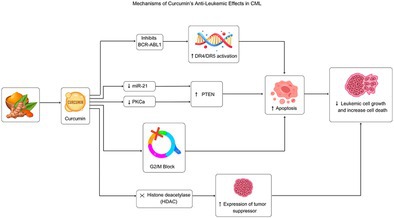

## 1 Introduction

Chronic myeloid leukemia (CML) is a hematologic malignancy characterized by the uncontrolled proliferation of myeloid cells due to the presence of the BCR‐ABL1 fusion gene, which results from the Philadelphia chromosome translocation (*t* (9; 22) (q34; q11)) (Jabbour and Kantarjian 2024; Kang et al. 2016; Sampaio et al. 2021). This fusion gene encodes a constitutively active tyrosine kinase that drives leukemogenesis by promoting aberrant cell signaling, leading to uncontrolled cell growth, resistance to apoptosis, and genomic instability (Chen et al. 2025; Yan et al. 2024). The advent of tyrosine kinase inhibitors (TKIs), such as imatinib, has significantly improved the prognosis of CML patients, transforming it from a fatal disease into a manageable condition (Jabbour and Kantarjian 2024). However, resistance to TKIs and the persistence of leukemic stem cells remain significant challenges, necessitating the exploration of novel therapeutic strategies (Costa et al. 2024).

Curcumin, a polyphenolic compound derived from *Curcuma longa* , has attracted considerable interest due to its broad spectrum of pharmacological properties, including anti‐inflammatory, antioxidant, and anticancer effects (Islam et al. 2024). Numerous studies have demonstrated curcumin's ability to modulate key molecular pathways involved in cancer progression, such as NF‐κB, PI3K/Akt, and JAK/STAT signaling, suggesting its potential role as an adjunctive therapy in various malignancies, including leukemia (Golmohammadi et al. 2024; Moon 2024; Zoi et al. 2024). In CML, curcumin has been reported to inhibit BCR‐ABL1 signaling, induce apoptosis, and overcome drug resistance, making it a promising candidate for further investigation (Meneses‐Sagrero et al. 2024; Morang et al. 2024).

This systematic review aims to summarize the current evidence regarding the anti‐leukemic effects of curcumin in CML. We will discuss its mechanisms of action, therapeutic potential, and possible limitations, highlighting its role as a complementary strategy alongside conventional CML treatments. Understanding the molecular basis of curcumin's effects may provide insights into new therapeutic approaches that could enhance treatment efficacy and overcome resistance in CML patients.

## 2 Methods

### 2.1 Protocol and Registration

This systematic review was conducted in accordance with the Preferred Reporting Items for Systematic Reviews and Meta‐Analyses (PRISMA) guidelines (Page et al. 2021). The study protocol was registered in the International Prospective Register of Systematic Reviews (PROSPERO) under registration number CRD42023479695.

### 2.2 Eligibility Criteria

#### 2.2.1 Inclusion Criteria

The inclusion criteria were defined using the Participants, Intervention, Comparator, and Outcomes (PICO) framework (Table 1). The in vitro studies using human CML cell lines were included to investigate the anticancer effects of curcumin or its analogs. Anticancer outcomes in in vitro studies included anti‐proliferative effects or growth inhibition, induction of apoptosis or enhanced sensitivity to TRAIL therapy, increased cytotoxicity against malignant cells, suppression of oncogenic signaling pathways, epigenetic regulation and transcriptional modulation, cell cycle arrest and mitotic disruption, regulation of miRNA expression, reduction in cell viability, and morphological changes indicative of cellular instability.

**TABLE 1:** Inclusion and exclusion criteria.

PICO	Inclusion criteria	Exclusion criteria
Patient	Human models of chronic myeloid leukemia (CML) cells	Animal models Human models not focused on CML cells Clinical studies
Intervention	Curcumin or its analogs	Studies examining curcumin in combination with other drugs or supplements
Comparison	All possible control groups (placebo or no treatment, standard care, observation)	Other study types (one‐armed/non‐controlled studies, case reports or series)
Outcome	All anticancer effects	Studies not assessing anticancer effects
Others	In vitro studies Language: English Full publication in a peer‐reviewed journal No publication year restriction	Gray literature (conference articles, abstracts, letters, ongoing studies, unpublished literature…) Reviews Book chapters

#### 2.2.2 Exclusion Criteria

Studies were excluded if they fell into any of the following categories: reviews, personal opinions, letters, congress abstracts, book chapters, or ongoing studies. Non‐English publications and animal studies were also excluded. Additionally, studies that investigated curcumin in combination with other drugs or supplements, as well as those unrelated to its antitumor effects on CML, were not considered. Trials conducted in non‐oncological clinical conditions or involving immunodeficient human models were excluded to maintain relevance to the study's focus. Finally, studies that did not include a control group were not eligible for inclusion.

### 2.3 Search Strategy

A systematic search was conducted in December 2024 across three electronic databases: PubMed, Scopus, and Web of Science. A comprehensive search strategy was developed for each database using a combination of MeSH terms, keywords, and text variations related to CML and curcumin therapy (Table S1). The following search terms were applied: (“curcumin” OR “turmeric” OR “curcuminoid” OR “diferuloylmethane” OR “ *curcuma longa* ” OR “mervia”) AND (“leukemia” OR “chronic myelogenous leukemia” OR “CML”) AND (“anti‐tumor” OR “apoptosis” OR “proliferation” OR “cytotoxic” OR “anti‐neoplastic” OR “cell migration” OR “cell cycle arrest” OR “cell death” OR “invasion” OR “anti‐cancer” OR “cell viability”). The search was designed to be highly sensitive, with no restrictions applied regarding study design or publication type. Additionally, the reference lists of relevant studies and prior reviews were manually screened to ensure that no eligible studies were overlooked.

### 2.4 Study Screening and Selection

Following the primary search, all retrieved citations were imported into EndNote 20 software, where duplicates were systematically removed. Two independent researchers (Z.S. and F.S.) conducted a two‐stage screening process based on the predefined eligibility criteria. In the first stage, the titles and abstracts of all identified articles were reviewed, and studies deemed relevant were selected for further evaluation. If a study provided insufficient information at this stage, it was retained for full‐text screening in the second stage. In the next stage, full‐text articles were assessed, and the inclusion and exclusion criteria were critically applied. Any disagreements between the reviewers were resolved through consensus or by consulting a third researcher (H.B.). Additionally, the reference lists of all retrieved articles were manually examined to identify any potentially relevant studies that had not been captured in the initial search.

### 2.5 Data Extraction

To minimize data collection bias, two independent researchers (Z.S. and L.K.) extracted key information from the included studies. The extracted data included authorship, year of publication, and country of study, cell line characteristics, type and dose of curcumin, duration of intervention, characteristics of the control group, main anticancer outcomes, and the proposed mechanism of action. Any discrepancies between the researchers were resolved through consensus or by consulting a third researcher (H.B.).

Due to substantial heterogeneity in study designs, interventions, and outcome measures, a meta‐analysis was not feasible. Instead, a narrative synthesis was conducted, without statistical or sensitivity analysis, in accordance with the Synthesis Without Meta‐analysis guideline (Campbell et al. 2020).

## 3 Results

### 3.1 Study Selection Process

**FIGURE 1:**
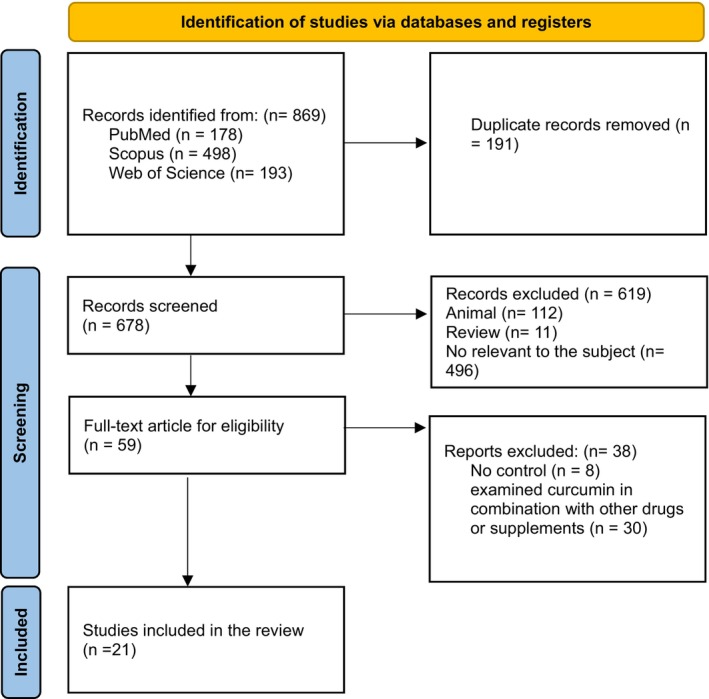
Flow chart of study selection for inclusion trials in the systematic review.

The initial search across PubMed, Scopus, and Web of Science identified 869 records (178 from PubMed, 498 from Scopus, and 193 from Web of Science). After removing 191 duplicates, 678 studies remained for screening. During the screening process, 619 studies were excluded based on predefined criteria: 112 focused on animal models, 11 were review articles, and 496 were irrelevant to the research topic. This left 59 full‐text articles for further review. Of these, 38 were excluded, 8 due to the lack of a control group, and 30 for investigating curcumin with other drugs or supplements. In the end, 21 studies met the inclusion criteria and were included in the final review (Figure 1).

### 3.2 Study Characteristics

The studies reviewed investigated curcumin's effects on CML using in vitro models. Researchers tested various formulations and concentrations of curcumin on leukemia cell lines and animal models to assess its therapeutic potential. Some studies used commercial‐grade curcumin, while others focused on modified derivatives to improve its bioavailability and effectiveness.

The leukemia cell lines tested included K562, HL‐60, Jurkat, LAMA84, REH, and MOLT‐4, with some studies also using patient‐derived leukemic cells for more clinical relevance. Curcumin concentrations in in vitro studies ranged from 0.05 to 50 μM, with treatment durations between 3 and 72 h. In animal models, curcumin was administered orally or by injection, with doses up to 20 mg/kg body weight.

Control conditions varied across studies, with some using untreated leukemia cells, others using vehicle‐treated controls (e.g., DMSO), or standard chemotherapy drugs like doxorubicin and etoposide. These conditions are fully detailed in Table 2.

**TABLE 2:** Characteristics of the included studies in the systematic review.

Author and year	Leukemia cell lines	Curcumin type	Curcumin dose	Course of treatment/duration of study	Control arm characteristic (type, dose, duration of treatment, etc.)	Anti‐leukemia outcomes (curcumin‐related outcomes on leukemic cells)	Mechanism of action
Roy et al. 2002	HL‐60, K‐562	Curcumin	—	24, 48 h	Control cells were treated with DMSO alone and positive controls with various amounts of doxorubicin	↓ Cell growth ↑ Apoptosis ↓ Cell proliferation ↓ Cell survival	↓ bcl‐2, an anti‐apoptotic gene ↑ p53 Activation ↑ Caspase‐3 and caspase‐8 ↓ PARP
Duvoix et al. 2003	K562, Jurkat	Curcumin	10 and 20 μM	24 h	Untreated cells cultured in 10% FCS medium	↑ Apoptosis in K562 and Jurkat cell lines, with Jurkat cells being more sensitive ↓ Pro‐caspase 8 and 9 levels and cleaved Bid in Jurkat cells ↓ Intact Bid levels in K562 cells	↑ Apoptosis via mitochondrial and receptor pathways ↓ Pro‐caspase 8 and 9 and activated the mitochondrial pathway by cleaving Bid. +I3:I7
Wu et al. 2003	K562, HL‐60	Curcumin	2.5, 5.0, 10 mg/L	24, 48 h	Etoposide (VP‐16), which has no influence on p210bcr/abl and has resistance to K562 cells, was used as an anticancer drug control to compare with curcumin > > 10 mg/L	↓ Cell growth ↓ Chemoresistance	↓ p210bcr/abl ↓ MEK‐1 ↓ c‐JUN
Anuchapreeda et al. 2006a	K562	Commercial grade curcumin (77% curcumin, 17% demethoxycurcumin, and 3% bisdemethoxycurcumin).	10 mM	48 h	Leukemic cells not treated with curcumin	↓ WT1 gene expression	↓ WT1 mRNA levels
Anuchapreeda et al. 2006b	ALL, AML, CML	Commercial grade curcumin (77% curcumin I, 17% curcumin II, and 3% curcumin III).	10 μM	48 h	Same cells as the intervention arm, but without curcumin treatment/78 patient leukemic cells (same cells, but without curcumin treatment)	↓ MDR1 gene expression ↑ Sensitivity to chemotherapy All results are statistically significant (*p* < 0.05)	↓ Pgp (encoded by MDR1)
Anuchapreeda et al. 2006c	K562	Commercial grade curcumin (77% curcumin, 17% demethoxycurcumin and 3% bisdemethoxycurcumin)	5, 10, or 15 μmol/L	2 or 3 days	K562 cells treated with vehicle control (0.01% DMSO) for 2 or 3 days	↓ WT1 protein and mRNA expression ↓ Cell proliferation with an IC₅₀ of 54 μmol/L, *p* < 0.05	↓ WT1 gene expression at both the mRNA and protein levels ↓ PKC activation
Chakraborty et al. 2006	K562	1,7‐bis‐(4‐hydroxy‐3‐methoxyphenyl)‐1,6‐heptadiene‐3,5‐dione	0, 1, 10, and 50 μM	24 and 48 h	Untreated K‐562 cells	↑ Caspase‐mediated apoptosis ↓ Telomerase activity	↑ Riggering the release of cytochrome c from mitochondria into the cytosol morphological changes (chromatin condensation, etc.) ↓ Sub‐G1 peak ↑ Release activates the caspase cascade, particularly caspase 3 and caspase 8 ↓ Bcl‐2 ↑ Bax. ↓ Telomerase activity ↓ TERT translocation to the nucleus
Jia et al. 2009	K562	Curcumin	20 mM	3–24 h	K562 leukemia cells treated with vehicle (DMSO)	↓ Cell viability with increasing dose and time ↑ Apoptosis ↑ Autophagy ↑ Cell death, (*p* < 0.05).	↑ Caspase‐3 ↓ Bcl‐2 ↑ Bid cleavage ↑ Autophagosome formation (LC3 immunoreactivity) ↑ LC3‐II protein ↑ Beclin 1
Semsri et al. 2011	K562	Pure curcumin	25 μM	48 h	0.04% DMSO	↓ WT1 expression ↓ PKCα activation ↓ WT1's auto‐regulation ↓ Proliferation, *p* < 0.05	↓ PKCα activation ↓ WT1 mRNA and protein levels
Chen et al. 2013	K562, HEL, 32D	Pure curcumin	20 μM	12 and 24 h	Untreated cells for each cell line Bone marrow mononuclear cells from healthy donors	↑ SOCS1 and SOCS3 expression ↓ Colony formation ↓ HDAC activity *p* < 0.01 ↓ Proliferation	↑ Acetylation of histones in the SOCS1 promoter regions ↑ Acetylation of histones in the SOCS3 promoter regions ↓ JAK2/STAT5 signaling
Gopal et al. 2014	JURKAT, K562, REH, MOLT‐4	Curcumin	0–50 μM	24–48 h	Normal human PBMCs (No treatment)	↓ Cell viability ↑ Apoptosis ↑ ROS generation ↓ Mitochondrial membrane potential ↓ Intracellular GSH *p* < 0.05	↑ Caspase‐3 and caspase‐9 activity
Taverna et al. 2015	K562 and LAMA84	Curcumin	5–40 μM/2 mg in mice	24 h		↓ Cell migration	↓ miR‐21 levels ↑ PTEN expression ↓ AKT phosphorylation ↓ VEGF expression release ↓ VEGF releasing ↓ Bcr‐Abl expression through the cellular ↑ miR‐196b
Lopes‐Rodrigues et al. 2017	K562 and K562Dox	Curcumin (1) and a novel curcumin derivative compound 10 (1,7‐bis(3‐methoxy‐4‐(prop‐2‐yn‐1‐yloxy)phenyl)hepta‐1,6‐diene‐3,5‐dione)	Curcumin (1): 20, 30, and 40 μM Compound 10: 2.7, 4.1, and 5.4 μM	48 h	K562 (sensitive chronic myeloid leukemia cell line)	↓ Cell growth ↑ G2/M phase arrest ↑ Apoptosis, *p* < 0.01	↑ p53 levels ↑ PARP‐1 cleavage ↓ Cyclin B1 levels ↓ Pro‐caspase 3 levels
Martinez‐Castillo et al. 2016	K562	Curcumin	20 μM	12, 18, and 24 h	K562 cells treated with 0.1% DMSO for 12, 18, or 24 h	↑ G2/M cell cycle arrest. ↑ Mitotic catastrophe ↑ Apoptosis, *p* < 0.05	↓ BCL‐2 ↓ XIAP protein levels ↓ P73 nuclear translocation by Transient NFκB activation ↓ C/EBPα expression. ↑ P73 protein levels
Taverna et al. 2016	K562 and LAMA84	Curcumin/curcu‐exosomes (20 and 50 μg/mL)	10, 20 and 40 μM/curcu‐Exo and Exo 100 μg in vivo	24 h	Control CML exosomes	↓ pro‐angiogenic proteins ↑ anti‐angiogenic proteins	↓ RhoB expression through miR‐21 transport ↓ Expression of VCAM1 at mRNA and protein levels with respect to control exosomes ↓ MARCKS expression
Lozada‐García et al. 2017	K562	Curcumin and 10 derivatives (compounds 2–11)	Compounds were evaluated at 50 μM, and the most active ones were further tested at 12.5 and 10 μM	48 h	Adriamycin used as a standard/control	↓ Cell growth IC50 values for selected compounds	↑ Methylation of the hydroxyl group
Martínez‐Castillo et al. 2018	HL‐60 and K562 cells	Curcumin	5, 10, 15, 20 or 30 μM	6, 12, 18, or 24 h	DMSO 0.1% (v/v) (curcumin vehicle) treatment for 24 h was used as a control culture in both cell lines	↓ Cellular viability ↑ Percentage of cell death ↑ Cell cycle arrest	↑ G2/M phase arrest in K562 cells ↑ The phosphorylated levels of Securin and BubR1 Caspases‐9 and ‐3 proteins activation (both processed and unprocessed forms)
Lestari et al. 2019	K562	A monocarbonyl analogue of curcumin. Pentagamavunon‐1 (PGV‐1)	(from 0.05 to 10 μM) of PGV‐1/curcumin and PGV‐1 (20 mg/kg BW) for mice	24 h, for 5 days	Curcumin (50 μM)	↑ Irreversible anti‐proliferative effect ↑ Tumor cell apoptosis with few side effects and a low risk of relapse ↑ Cell senescence ↑ Cell death	↑ Prometaphase arrest in the M phase of the cell cycle ↑ Intracellular ROS levels ↓ ROS‐metabolic enzymes ↑ PGV‐1
Surapally et al. 2020	HL60, K562, MOLT4, KG1	Curcumin	5, 10, 25, and 50 μM	24 h	Untreated controls	↑ TRAIL‐induced apoptosis ↓ Cell viability by The IL2‐TRAIL peptide and curcumin combination	↑ TRAIL‐apoptotic signaling in leukemic cells ↑ Expression of DR4 and DR5 ↓ cFLIP and anti‐apoptotic proteins Mcl‐1, Bcl‐xl, and XIAP
Bilajac et al. 2022	K562, LAMA84S, LAMA84R	Natural polyphenol derived from turmeric	10–30 μM for K562 and LAMA84S, 25–200 μM for LAMA84R	48 h	0.1% DMSO for 48 h	↓ Cell viability ↓ Proliferation ↑ Cell death, *p* < 0.05	↓ p‐NF‐κB activity ↓ p‐Akt ↓ p‐P70S6K expression ↑ Expression of caspase‐3
Feriotto et al. 2023	K562	Isoxazole curcumin derivatives (2 and 22) and oxime curcumin derivative (128)	0.5, 12.1 μM (derivatives 2 and 22, derivative 128)	72 h	K562 cells treated with DMSO (vehicle control)	↑ Cytotoxicity ↑ Mitochondrial dysfunction. ↑ Reversing drug resistance ↑ Cell cycle arrest ↑ Apoptosis, *p* < 0.05	↑ Caspase activation ↓ BCR‐ABL expression

Abbreviations: ALL, acute lymphoblastic leukemia; AML, Acute Myeloid Leukemia; Bax, Bcl‐2‐associated X protein; Bcl‐2, B‐cell lymphoma 2; Bcl‐xl, B‐cell lymphoma‐extra large; c/ebpα, CCAAT enhancer binding protein alpha; cFLIP, cellular FLICE‐like inhibitory protein; CML, Chronic Myelogenous Leukemia; DMSO, dimethyl sulfoxide; DR4, death receptor 4; DR5, death receptor 5; FACS, Fluorescence‐activated cell sorting; FCS, fetal calf serum; GSH, Glutathione; HDAC, Histone deacetylase; HUVEC, Human Umbilical Vein Endothelial Cell; IC50, Half‐maximal inhibitory concentration; IL‐2, Interleukin 2; JAK2, Janus Kinase 2 gene; MARCKS, Myristoylated Alanine Rich Protein Kinase C Substrate; MCL‐1, myeloid leukemia 1; MDR1, Multidrug Resistance Mutation; MEK‐1, Mitogen‐Activated Protein Kinase Kinase 1; miR‐196b, microRNA‐196b; miR‐21, microRNA‐21; MPN, myeloproliferative neoplasms; NF‐κB, nuclear factor kappa‐light‐chain‐enhancer of activated B cells; p‐AKT, phosphorylated AKT; PARP‐1, poly [ADP‐ribose] polymerase 1; PBMC, Primary Peripheral Blood Mononuclear Cells; P‐gp, P‐glycoprotein; PKC, protein kinase C; PKCα, Protein Kinase C α; p‐P70S6K, phospho‐p70 S6 kinase; PTEN, phosphatase and tensin homolog; RhoB, Ras Homolog Family Member B; ROS, reactive oxygen species; SOCS1, suppressor of cytokine signaling 1; SOCS3, suppressor of cytokine signaling 3; STAT5, Signal Transducer and Activator of Transcription; TERT, telomerase reverse transcriptase; TRAIL¸TNF‐related apoptosis‐inducing ligand; VCAM1, vascular cell adhesion molecule 1; VEGF, vascular endothelial growth facto; WT1, Wilms Tumor 1; XIAP, X‐linked inhibitor of apoptosis.

### 3.3 Curcumin's Anti‐Leukemic Effects

#### 3.3.1 Anti‐Proliferative Properties

Curcumin showed strong anti‐proliferative effects in leukemia cells, especially in CML (K562) and AML (HL‐60) cell lines. The inhibition of growth was dose‐dependent, with curcumin proving more effective against K562 cells than other leukemia models (Anuchapreeda et al. 2006b; Bilajac et al. 2022; Roy et al. 2002; Wu et al. 2003). Curcumin also inhibited PKCα signaling, which reduced WT1 expression in K562 cells, further enhancing its anti‐proliferative effect (Anuchapreeda et al. 2006a; Semsri et al. 2011).

#### 3.3.2 Modulation of Oncogenic Signaling Pathways

A key mechanism underlying curcumin's anti‐cancer effects was its ability to downregulate oncogenic signaling pathways. Specifically, curcumin reduced p210 BCR‐ABL expression in K562 cells, a hallmark of CML. Additionally, it downregulated MEK‐1 and c‐JUN proteins, further disrupting leukemic cell survival and proliferation (Wu et al. 2003).

#### 3.3.3 Epigenetic Regulation and Transcriptional Modulation

Curcumin was also found to influence epigenetic regulation by inhibiting histone deacetylases (HDACs), leading to increased expression of tumor‐suppressor genes such as SOCS1 and SOCS3. Additionally, silencing HDAC8 enhances curcumin's effect on upregulating these genes, highlighting its role in gene expression control (Chen et al. 2013).

#### 3.3.4 MicroRNA (miRNA) Regulation

Curcumin affected miRNA expression profiles in leukemia cells, notably reducing miR‐21 levels while increasing PTEN expression. In addition, the upregulation of miR‐196b led to the downregulation of BCR‐ABL, a critical factor for the survival of leukemic cells in CML (Taverna et al. 2016, 2015).

#### 3.3.5 Induction of Apoptosis and Sensitization to TRAIL Therapy

Several studies showed that curcumin can trigger apoptosis by increasing the levels of death receptors DR4 and DR5 and mitochondrial pathways (Duvoix et al. 2003; Gopal et al. 2014). This effect was especially strong when combined with IL2‐TRAIL peptide therapy, leading to near‐complete killing of leukemia cells (Chakraborty et al. 2006; Surapally et al. 2020). Additionally, curcumin has been associated with autophagy induction, as evidenced by increased LC3‐II and beclin‐1 protein levels and the formation of autophagosomes (Jia et al. 2009).

#### 3.3.6 Cell Cycle Arrest and Mitotic Disruption

Curcumin also impacted cell cycle regulation, especially by causing G2/M phase arrest in K562 cells. This arrest led to faulty mitotic spindle formation, triggering apoptosis via a p73‐dependent pathway (Lopes‐Rodrigues et al. 2017; Martinez‐Castillo et al. 2016; Martínez‐Castillo et al. 2018).

#### 3.3.7 Curcumin Derivatives and Their Enhanced Anti‐Cancer Potential

In addition to natural curcumin, several studies investigated curcumin derivatives, like compounds 2 and 10. These derivatives were more cytotoxic than natural curcumin and were especially effective at inhibiting P‐glycoprotein (P‐gp), helping to overcome multidrug resistance in leukemia cells (Anuchapreeda et al. 2006c; Feriotto et al. 2023; Lopes‐Rodrigues et al. 2017; Lozada‐García et al. 2017).

#### 3.3.8 Comparative Analysis With PGV‐1

PGV‐1, a curcumin analog, demonstrated superior anti‐tumor activity. PGV‐1 induced prometaphase arrest and ROS‐mediated cell death with minimal side effects (Lestari et al. 2019).

### 3.4 Curcumin as a Potential Adjunct in Leukemia Treatment: Enhancing Efficacy and Overcoming Drug Resistance

**FIGURE 2:**
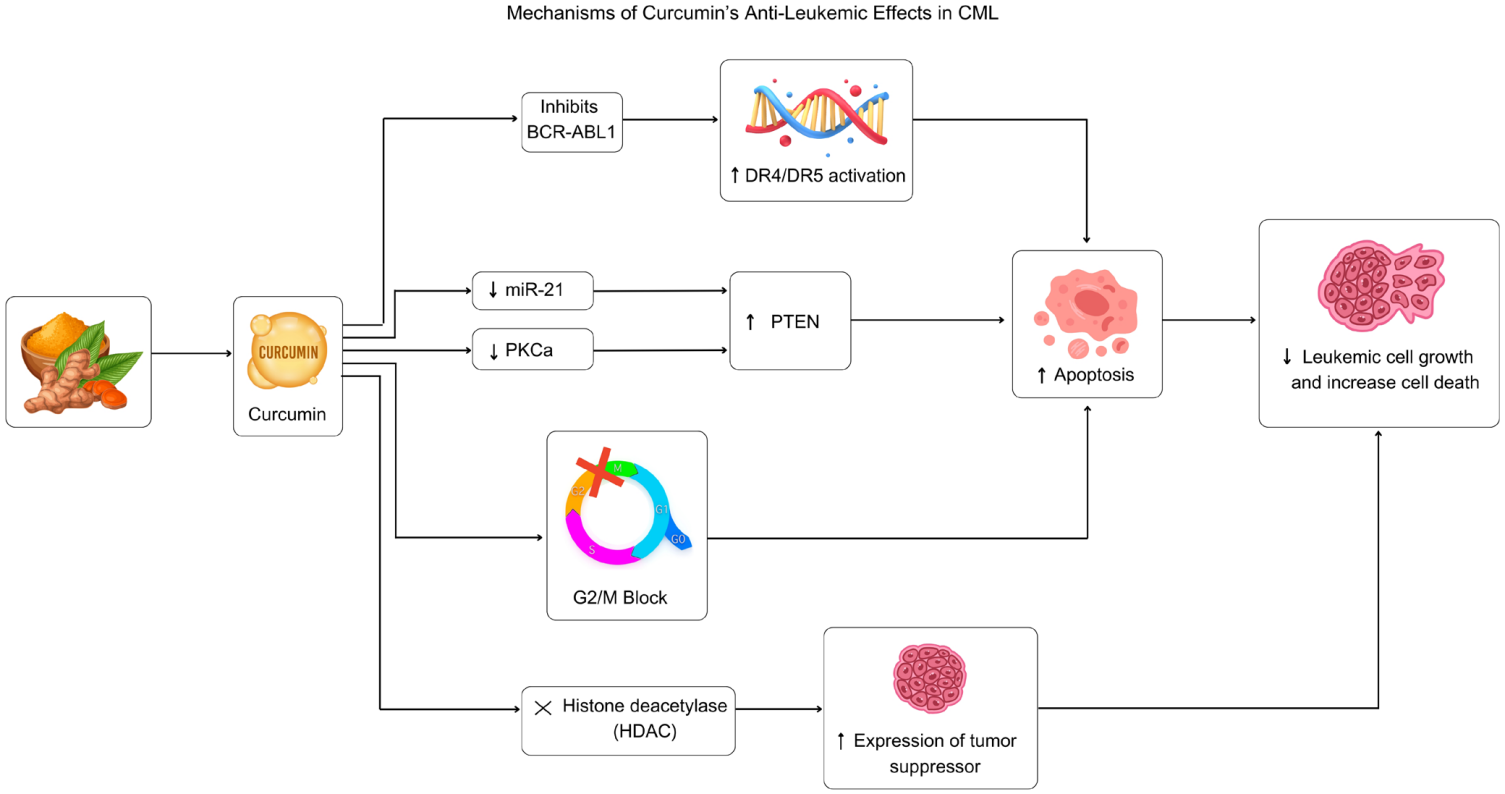
Curcumin exerts anti‐leukemic effects in chronic myeloid leukemia (CML) by inhibiting breakpoint cluster region–Abelson murine leukemia viral oncogene homolog 1 (BCR‐ABL1), modulating microRNA‐21 (miR‐21), protein kinase C alpha (PKCα), and phosphatase and tensin homolog (PTEN), activating death receptor 4/5 (DR4/DR5), blocking G2/M cell cycle progression, and inhibiting histone deacetylase (HDAC), leading to apoptosis and suppression of leukemic cell proliferation.

Several studies suggest that curcumin could play a key role in combination therapies for leukemia, especially when it comes to overcoming drug resistance and boosting treatment effectiveness. Wu et al. found that curcumin inhibited leukemia cell proliferation and reversed chemoresistance (Wu et al. 2003), while Surapally et al. demonstrated its role in enhancing TRAIL‐induced apoptosis (Surapally et al. 2020). Lopes‐Rodrigues et al. showed that curcumin and its derivative improved apoptosis and inhibited P‐gp in drug‐resistant CML cells (Lopes‐Rodrigues et al. 2017). Additionally, Feriotto et al. reported that curcumin derivatives effectively induced apoptosis and reversed resistance in imatinib‐resistant K562 cells (Feriotto et al. 2023). These findings suggest curcumin's potential as a complementary anti‐leukemic agent (Figure 2).

## 4 Discussion

This systematic review highlights the potential therapeutic effects of curcumin in leukemia, particularly CML, based on 21 selected in vitro and in vivo studies. The included studies provide compelling evidence of curcumin's anti‐leukemic properties, including its ability to inhibit cell proliferation, modulate oncogenic signaling, regulate gene expression, induce apoptosis, and overcome drug resistance (Bilajac et al. 2022). The findings suggest that curcumin, either alone or in combination with existing therapies, could be a promising adjunct in leukemia treatment (Wu et al. 2002).

A consistent finding across the studies is curcumin's potent anti‐proliferative effect on leukemia cell lines, particularly K562 (CML) and HL‐60 (AML). The anti‐proliferative action was dose dependent and was more pronounced in CML models. Mechanistically, curcumin inhibited the PKCα pathway and subsequently reduced WT1 expression, contributing to decreased leukemic cell growth (Anuchapreeda et al. 2006a; Anuchapreeda et al. 2006b; Anuchapreeda et al. 2006c).

One of curcumin's key anti‐leukemic mechanisms involves the modulation of oncogenic signaling. Specifically, curcumin downregulated the p210 BCR‐ABL fusion protein—a hallmark of CML—and suppressed downstream signaling proteins such as MEK‐1 and c‐JUN, effectively impairing leukemic cell survival. In parallel, curcumin influenced epigenetic regulators by inhibiting HDACs, particularly HDAC8, which led to the upregulation of tumor suppressor genes such as SOCS1 and SOCS3 (Wu et al. 2003) (Chen et al. 2013).

Recent studies have demonstrated that curcumin exerts potent anti‐leukemic effects through multiple mechanisms, including the induction of apoptosis and modulation of key signaling pathways. In K562 cells, curcumin induces apoptosis by inhibiting telomerase activity and activating caspases‐3 and ‐8, leading to mitochondrial dysfunction and increased cytochrome c release (Chakraborty et al. 2006). It also causes cell cycle arrest at the G0/G1 phase, enhancing apoptotic responses through the regulation of Bax, Bcl‐2, and caspase‐3 expression (Li et al. 2022).

Additionally, curcumin sensitizes CML cells to oxidative stress by downregulating nuclear factor erythroid 2‐related factor 2 (Nrf2), thus amplifying apoptosis (Larasati et al. 2018).

### 4.1 Curcumin Derivatives and Combination Strategies

Investigations into curcumin derivatives, particularly C1206, have revealed enhanced anti‐leukemic activity, primarily through inhibition of heat shock protein 90 (Hsp90) in CML models (Fan et al. 2018). Curcumin also activates death receptors DR4 and DR5, enhances mitochondrial‐mediated apoptosis by modulating Bcl‐2 family proteins, and demonstrates efficacy when combined with IL2‐TRAIL peptide therapy (Bahadar et al. 2024; Duvoix et al. 2003; Shankar et al. 2007; Surapally et al. 2020). Additionally, it induces autophagy, as evidenced by increased levels of LC3‐II and beclin‐1, suggesting a dual mechanism of apoptosis and autophagy underlying its cytotoxic activity in leukemia (Jia et al. 2009; Khan et al. 2012).

### 4.2 Curcumin in Solid Tumors and Broader Anti‐Cancer Potential

Beyond hematologic malignancies, curcumin's apoptotic potential is evident in several solid tumors. Najafi et al. demonstrated that co‐administration of curcumin with chemotherapeutic agents in gastric cancer significantly elevated apoptotic markers and diminished chemoresistance, primarily via the inhibition of NF‐κB signaling, a key mediator in inflammation and cell survival (Najafi et al. 2020). This finding, derived from a review of 13 preclinical studies, supports Level I evidence that curcumin serves as an effective adjuvant, enhancing the cytotoxicity of standard treatments.

Curcumin's capacity to modulate oxidative stress and microRNA signaling further underscores its therapeutic versatility. Ding et al. revealed that curcumin synergizes with bortezomib in multiple myeloma by affecting apoptotic signaling pathways, thereby improving drug response and reducing resistance (Ding et al. 2022). Additionally, Liu et al. identified a novel ROS/KEAP1/NRF2 pathway through which curcumin modulates the expression of miR‐34a/b/c in colorectal cancer (CRC) cells, subsequently reducing metastatic potential and enhancing apoptosis (Liu et al. 2023).

This interplay between curcumin, oxidative stress, and epigenetic regulation suggests a unifying mechanism behind its broad‐spectrum anti‐cancer effects. Collectively, these findings advocate for further clinical exploration of curcumin as a multi‐targeted therapeutic and adjuvant in oncology.

### 4.3 miRNA Regulation and Mechanistic Insights in CML

Several studies in the review also demonstrate curcumin's role in regulating miRNAs, particularly miR‐21, a key player in the pathophysiology of CML. Curcumin has been shown to downregulate miR‐21 expression, thereby facilitating the upregulation of PTEN, a tumor suppressor gene, which in turn contributes to the inhibition of leukemic cell survival (Taverna et al. 2016).

This is particularly significant given the robust data identifying miR‐21 as one of the most overexpressed miRNAs in CML patients. Parsa‐Kondelaji et al. reported a mean fold increase of 9.22 in miR‐21 expression compared to controls, with levels escalating through disease progression, from 7.16 in the chronic phase to 13.20 in blast crisis, highlighting miR‐21's utility as both a biomarker and a pathological mediator (Parsa‐Kondelaji et al. 2023).

Furthermore, curcumin's upregulation of miR‐196b has been associated with suppression of BCR‐ABL expression, enhancing its therapeutic relevance in CML. Given miR‐21's involvement in activating oncogenic pathways such as PI3K/AKT (Wang et al. 2015), and its established role in chemoresistance (Lahlil et al. 2023; Zhang et al. 2021), curcumin's modulation of this miRNA underscores its potential in overcoming drug resistance.

Taverna et al. further showed that curcumin alters miR‐21 in CML‐derived exosomes, influencing angiogenesis, while Seca et al. confirmed that targeting miR‐21 improves chemosensitivity (Seca et al. 2013; Taverna et al. 2016).

Altogether, these findings position miRNA modulation—especially of miR‐21—as a promising adjunctive strategy in curcumin‐based CML therapy.

### 4.4 Mechanisms of Cell Cycle Arrest and Resistance Reversal

The ability of curcumin to induce cell cycle arrest, particularly at the G2/M phase, further underscores its potential as an anti‐leukemic agent (Martinez‐Castillo et al. 2016; Martínez‐Castillo et al. 2018). By disrupting mitotic spindle formation and triggering apoptosis via a p73‐dependent pathway, curcumin effectively inhibits the proliferation of leukemia cells (Ahmad et al. 2017; Martínez‐Castillo et al. 2018).

Moreover, studies on curcumin derivatives have demonstrated that structural modifications enhance its potency, particularly through the inhibition of P‐gp, thereby overcoming multidrug resistance, one of the major obstacles in leukemia therapy (Lopes‐Rodrigues et al. 2017).

### 4.5 Comparative Studies With Standard Leukemia Treatments

Several studies have highlighted curcumin's potential as an adjunctive agent alongside standard chemotherapeutics in leukemia treatment. In CML, curcumin has demonstrated comparable efficacy to tyrosine kinase inhibitors (TKIs), such as imatinib, through downregulation of the BCR‐ABL oncoprotein (Wu et al. 2003). This supports its role in overcoming drug resistance, particularly in imatinib‐refractory cases. Curcumin also enhances the efficacy of TRAIL‐based therapies and sensitizes resistant CML cells by targeting key survival pathways (Feriotto et al. 2023; Surapally et al. 2020). Furthermore, its ability to inhibit P‐gp, a major mediator of multidrug resistance, reinforces its utility in overcoming chemotherapy failure (Lopes‐Rodrigues et al. 2017).

### 4.6 Insights From Other Leukemia Types (AML and ALL)

Beyond CML, curcumin has demonstrated notable effects in other leukemia models. In AML cells, curcumin inactivates the AKT pathway, triggering both cell cycle arrest and apoptosis (Zhou et al. 2021). Zahed Panah et al. found that curcumin significantly improved the growth‐inhibitory effect of daunorubicin in primary CD34+/CD38− AML cells, largely through the downregulation of osteopontin (OPN), a gene associated with leukemic stem cell survival and chemoresistance via the AKT/mTOR pathway (Ahmed et al. 2020).

Furthermore, Kermanshahi et al. demonstrated curcumin's ability to enhance the therapeutic index of cytarabine while suppressing MDR gene expression, including MDR1, LRP, and BCRP, in both wild‐type FLT3 and FLT3‐ITD AML models (Ahmed et al. 2020). Ahmed et al. also observed that curcumin, in combination with other phytochemicals, selectively induced apoptosis in AML cells while sparing healthy peripheral blood mononuclear cells, suggesting its safety and specificity. These findings collectively underscore curcumin's potential in attenuating drug resistance and improving therapeutic outcomes in AML (Trachtenberg et al. 2019).

In a similar vein, Haghighian et al. reported that curcumin upregulates p53 and downregulates NF‐κB in acute lymphoblastic leukemia (ALL) cells, thus amplifying apoptotic responses when used in conjunction with therapies such as L‐asparaginase (Khadem Haghighian et al. 2020).

These findings underscore curcumin's broader potential across various hematologic malignancies by targeting key apoptotic regulators and enhancing treatment efficacy.

### 4.7 Limitations and Future Research

Despite promising preclinical evidence supporting curcumin's therapeutic potential in leukemia—particularly in CML and AML—several important limitations must be addressed before clinical translation is feasible. A major challenge lies in curcumin's inherently low systemic bioavailability due to its poor water solubility, rapid metabolism, and limited absorption. This pharmacokinetic barrier significantly compromises its therapeutic effectiveness in vivo.

To address this, researchers have developed a variety of advanced delivery methods. Nanoformulations, such as polymeric nanoparticles, lipid‐based carriers, and micelles, have improved their solubility and stability. Liposomal systems and exosome‐based delivery approaches have also enhanced cellular uptake and prolonged their therapeutic effects. Additionally, chemically modifying curcumin or creating prodrugs has shown promise in boosting their activity and metabolic resilience (Lestari et al. 2019).

However, there are still gaps in the research landscape. Many preclinical studies differ widely in their design, dosages, treatment durations, and model systems, making it difficult to compare results or draw consistent conclusions. Moreover, most of the existing evidence comes from lab‐based or animal studies, with limited support from large‐scale clinical trials in humans.

Moving forward, future research should prioritize three key areas:

1. Standardizing preclinical methods to improve consistency across studies;
2. Conducting robust clinical trials to evaluate curcumin's safety and effectiveness in real‐world settings; and
3. Refining delivery technologies to overcome bioavailability issues and maximize therapeutic benefit.

By addressing these issues, we can bring curcumin closer to becoming a reliable and effective option in leukemia treatment.

## 5 Conclusions

This systematic review highlights the promising anti‐leukemic potential of curcumin and its derivatives in the treatment of CML. The in vitro evidence demonstrates that curcumin exerts significant cytotoxic effects on CML cell lines, primarily through the induction of apoptosis, cell cycle arrest, autophagy, and the disruption of key oncogenic signaling pathways, including BCR‐ABL1, PKCα, WT1, and HDAC8. Moreover, curcumin modulates critical regulatory molecules such as miR‐21, PTEN, and SOCS1/3, further contributing to its anti‐proliferative effects. Importantly, synthetic curcumin analogs such as PGV‐1 and C1206 show enhanced efficacy and the ability to overcome TKI resistance, suggesting potential for therapeutic application in drug‐resistant CML.

Despite these encouraging findings, the translational application of curcumin is limited by its poor bioavailability and lack of clinical data. Future studies should prioritize the development of bioavailable formulations and conduct in vivo validations to bridge the gap between preclinical efficacy and clinical application. Overall, curcumin represents a compelling adjunct candidate to conventional CML therapies and warrants further investigation within integrative treatment strategies.

## Author Contributions

**Hossein Bahari:** conceptualization (equal), methodology (equal), project administration (equal), writing – original draft (equal). **Zeinab Salim:** data curation (equal), investigation (equal). **Leila Kardanpour:** data curation (equal), investigation (equal). **Hosein Kouchaki:** writing – review and editing (equal). **Fatemeh Shoja:** data curation (equal), investigation (equal). **Iman Rahnama:** software (equal), visualization (equal). **Arezoo Faridzadeh:** validation (equal), writing – original draft (equal), writing – review and editing (equal). **Maryam Ranjbar Zahedani:** supervision (equal).

## Ethics Statement

The authors have nothing to report.

## Consent

The authors have nothing to report.

## Supporting information


**Table S1:** Search strategy.

## Data Availability

Data will be available upon reasonable request from the authors.
