# Supplementary material for: The Anti‐Leukemic Potential of Curcumin in Chronic Myeloid Leukemia: A Systematic Review of In Vitro Studies
Source: Food Sci Nutr. 2025 Sep 7;13(9):e70852. doi: 10.1002/fsn3.70852 (PMC12415269; doi:10.1002/fsn3.70852)
Supplement: Supplementary file 1 — Table S1: Search strategy. [file FSN3-13-e70852-s001.docx]

**Supplementary Table 1. Search strategy**

| **Search strategy** | **Database** |
| --- | --- |
| **1#**  "leukemia"[MeSH] OR "Chronic Myelogenous Leukemia"[TW] OR "leukemic cells"[TW] OR "CML"[TW]  **#2**  "Curcumin"[MeSH] OR "Curcuma longa"[TW] OR "turmeric"[TW] OR "diferuloylmethane"[TW] OR "Curcuma longa extract"[TW] OR "curcuminoids"[TW] OR "Curcumin Phytosome"[TW] OR "curcumin analog*"[TW]  **#3**  "cell proliferation"[MeSH] OR "apoptosis"[MeSH] OR "antitumor"[TW] OR "anti-neoplastic"[TW] OR "cytotoxic"[TW] OR "cell death"[TW] OR "proliferation"[TW] OR "anti-cancer"[TW] OR "metastasis"[TW] OR "toxicity" OR "cell migration"[TW] OR "invasion"[TW] OR "viability"[TW] OR "tumor volume"[TW] OR "tumor size"[TW] OR "cell cycle arrest"[TW] OR "Cell Multiplication"[TW] OR "angiogenesis"[TW] OR "anti-proliferative activity"[TW]  **4# 1# AND 2# AND 3#** | PubMed    (n: 178) |
| **1#**  TITLE-ABS-KEY("leukemia") OR TITLE-ABS-KEY("Leukemia cells") OR TITLE-ABS-KEY("Chronic myelogenous Leukemia") OR TITLE-ABS-KEY("CML")  **2#**  TITLE-ABS-KEY("curcumin") OR TITLE-ABS-KEY("nanocurcumin") OR TITLE-ABS-KEY("Turmeric") OR TITLE-ABS-KEY("curcuminoid formulations") OR TITLE-ABS-KEY("Curcuma longa") OR TITLE-ABS-KEY("Curcuma longa extract") OR TITLE-ABS-KEY("Mervia") OR TITLE-ABS-KEY("Diferuloylmethane") OR TITLE-ABS-KEY("Corcuminoids") OR TITLE-ABS-KEY("Curcuminoid of turmeric") OR TITLE-ABS-KEY("Curcumin Phytosome") OR TITLE-ABS-KEY("curcumin analog")  **3#**  TITLE-ABS-KEY(anti-tumor) OR TITLE-ABS-KEY(anti-proliferative activity) OR TITLE-ABS-KEY("apoptosis") OR TITLE-ABS-KEY("anti-neoplastic") OR TITLE-ABS-KEY("cytotoxic") OR TITLE-ABS-KEY("anti-cancer") OR TITLE-ABS-KEY("cell death") OR TITLE-ABS-KEY("invasion") OR TITLE-ABS-KEY("proliferation") OR TITLE-ABS-KEY("angiogenesis") OR TITLE-ABS-KEY("Anti proliferation") OR TITLE-ABS-KEY("anti-neoplastic") OR TITLE-ABS-KEY("metastasis") OR TITLE-ABS-KEY("toxicity") OR TITLE-ABS-KEY("migration") OR TITLE-ABS-KEY("cell viability") OR TITLE-ABS-KEY("tumor volume") OR TITLE-ABS-KEY("tumor size") OR TITLE-ABS-KEY("cell cycle arrest") OR TITLE-ABS-KEY("Cell Multiplication")  **4# 1# AND 2# AND 3#** | Scopus  (n: 498) |
| **1#**  TS ("leukemia") OR TS=( "Leuaekemia") OR TS=("Chronic myelogenous leukemia") OR TS=("CML")  **2#**  TS=("Curcumin") OR TS=("turmeric extract") OR TS=("diferuloylmethane") OR TS=("curcuminoid")  **3#**  TS=("antitumor") OR TS=("antineoplastic") OR TS=("anticancer") TS=("angiogenesis") OR TS=("tumor inhibition") OR TS=("cancer suppression")  **4# 1# AND 2# AND 3#** | Web of science  (n: 193) |
